# Supplementary material for: Deciphering the Role of Leptospira Surface Protein LigA in Modulating the Host Innate Immune Response
Source: Front Immunol. 2021 Dec 16;12:807775. doi: 10.3389/fimmu.2021.807775 (PMC8716722; doi:10.3389/fimmu.2021.807775)
Supplement: Supplementary file 1 [file DataSheet_1.docx]

**Supplementary Materials**

**Title:** Deciphering the role of *Leptospira* surface protein LigA in modulating the host innate immune response

Authors: Ajay Kumar ,Vivek P. Varma, Kavela Sridhar , Mohd Abdullah, Pallavi Vyas, Muhammed Ashiq T, Yung-Fu Chang, Syed M. Faisal^*^

*Correspondence to: [faisal@niab.org.in](mailto:faisal@niab.org.in) or smfaisal77@gmail.com

**The supplementary file contains-**

**Figure S1-** Purification of recombinant proteins.

**Figure S2-** Effect on TLR activity after pre-treatment of purified recombinant proteins with Polymixin B and Proteinase K

**Figure S3-** Circular dichroism spectra of the recombinant proteins.

**Figure S4-** Bacterial survival assay

**Table 1-** Primers used for RT-PCR

**Table 2-** Primers used for creating deletion domains of LAV

**
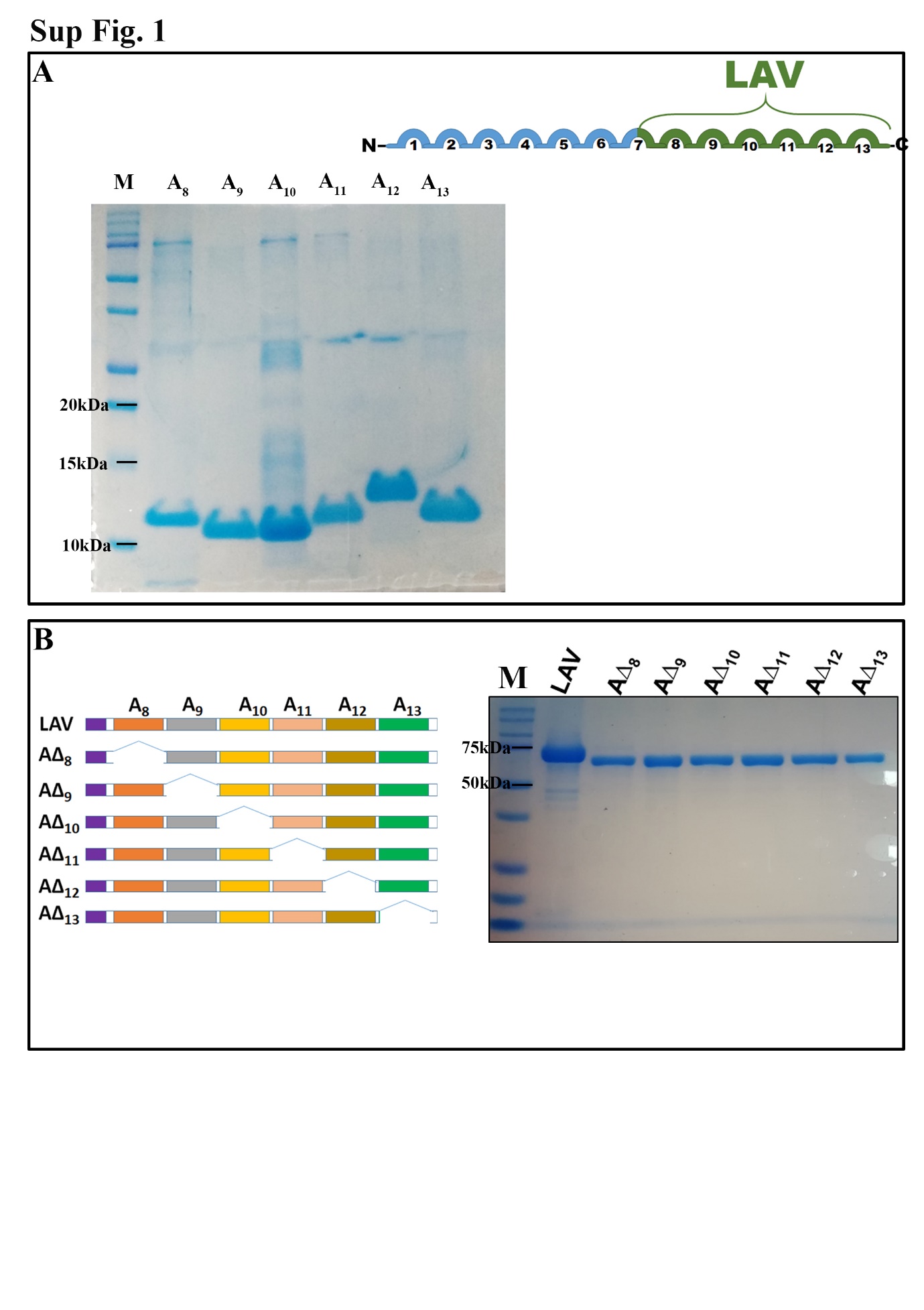
Figure S1**

**Sup. Fig. 1: Purification of recombinant proteins.**

**(A)** *SDS PAGE profile of domains of Variable A (LAV).* The recombinant proteins were purified as His-sumo fusion proteins as described in materials and methods. The expected molecular weight of each domain ranges from 11-12kd . **(B)** *Schematic presentation of strategy of creating LAV domain deletion mutants (A∆_8_- A∆_13_) by PCR based site-directed mutagenesis and SDS PAGE profile of purified proteins.*  The recombinant proteins were purified as His-sumo fusion proteins as described in materials and methods The expected molecular weight of LAV was 73kd and each domain deletion mutant was ~63kd. Data are representative of three independent experiments. (*Indicates P < 0.05).

**Figure S2**

**
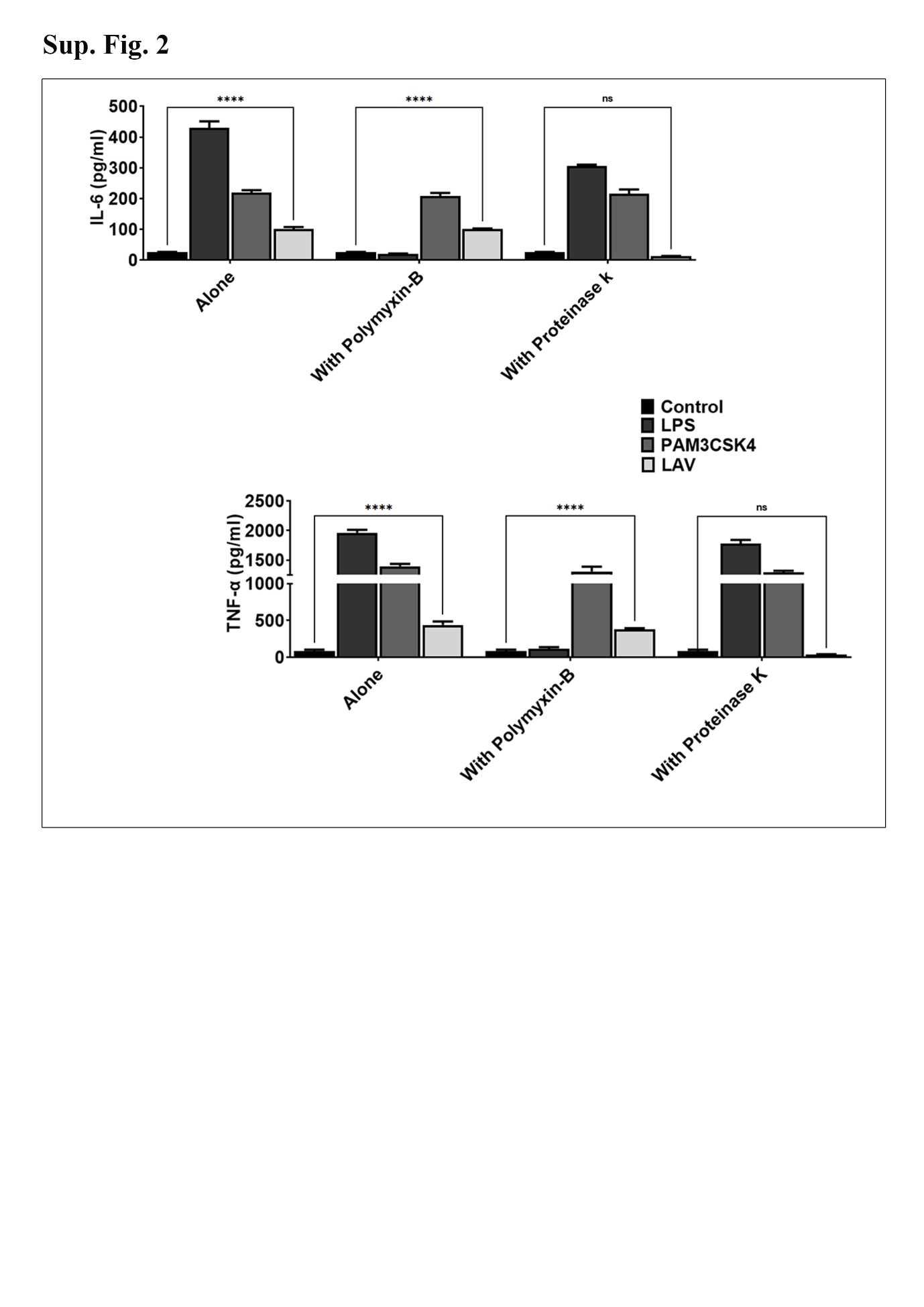
**

**Sup. Fig. 2: Effect on TLR activity after pre-treatment of purified recombinant proteins with Polymixin B and Proteinase K.** RAW264.7 cells were incubated with 2μg/ml of purified LAV pre-treated with Polymyxin B or Proteinase- K as mentioned in materials and methods and supernatant was collected to measure levels of IL-6 and TNF-α by ELISA.

**Figure S3**

**
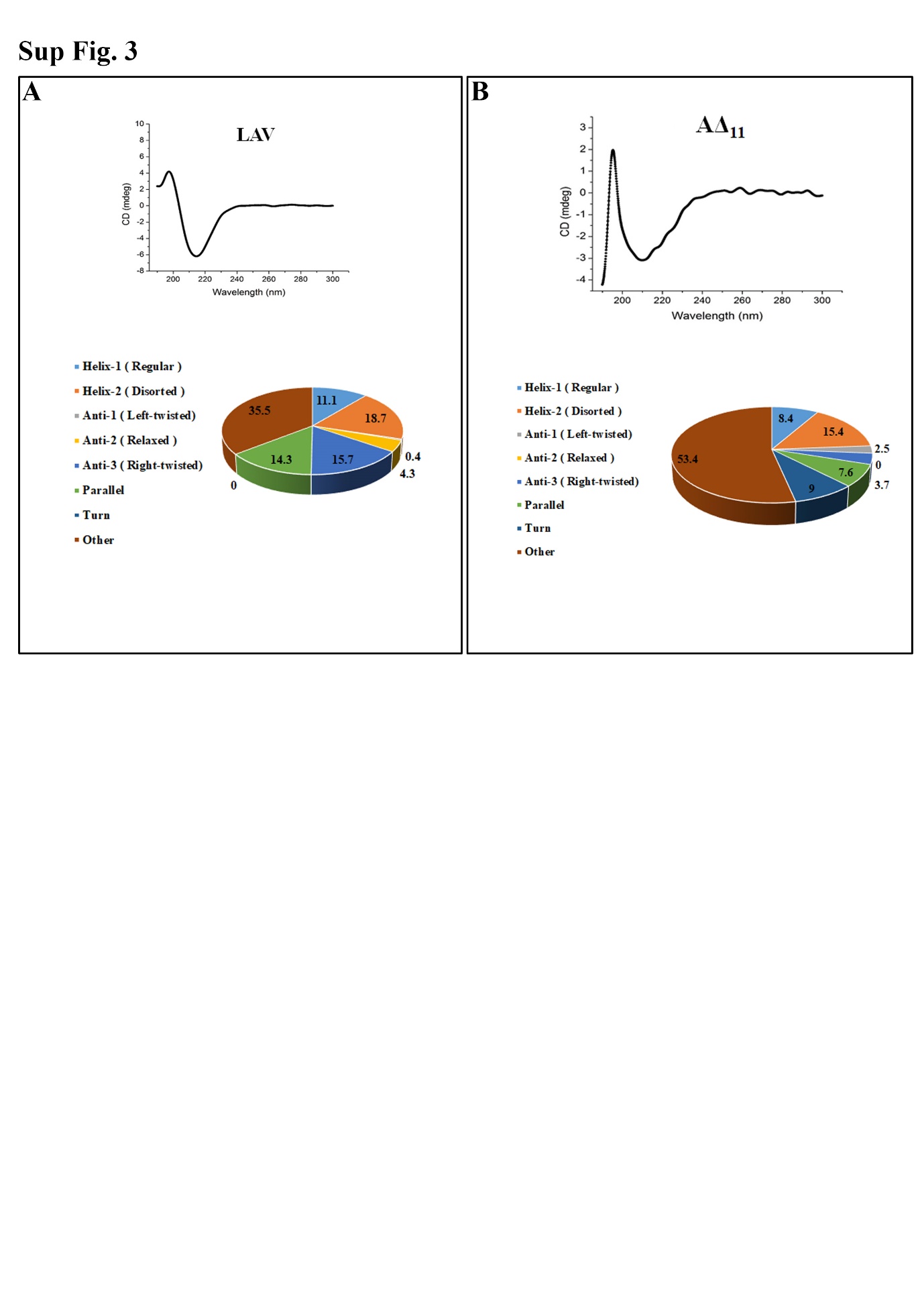
**

**Sup Fig. 3** **Circular dichroism spectra of the recombinant proteins***.* CD spectra of recombinant proteins LigA WT and LigA∆11. Far-UV CD spectra are presented as an average of five scans recorded from 190 to 300 nm.

**Figure S4**

**
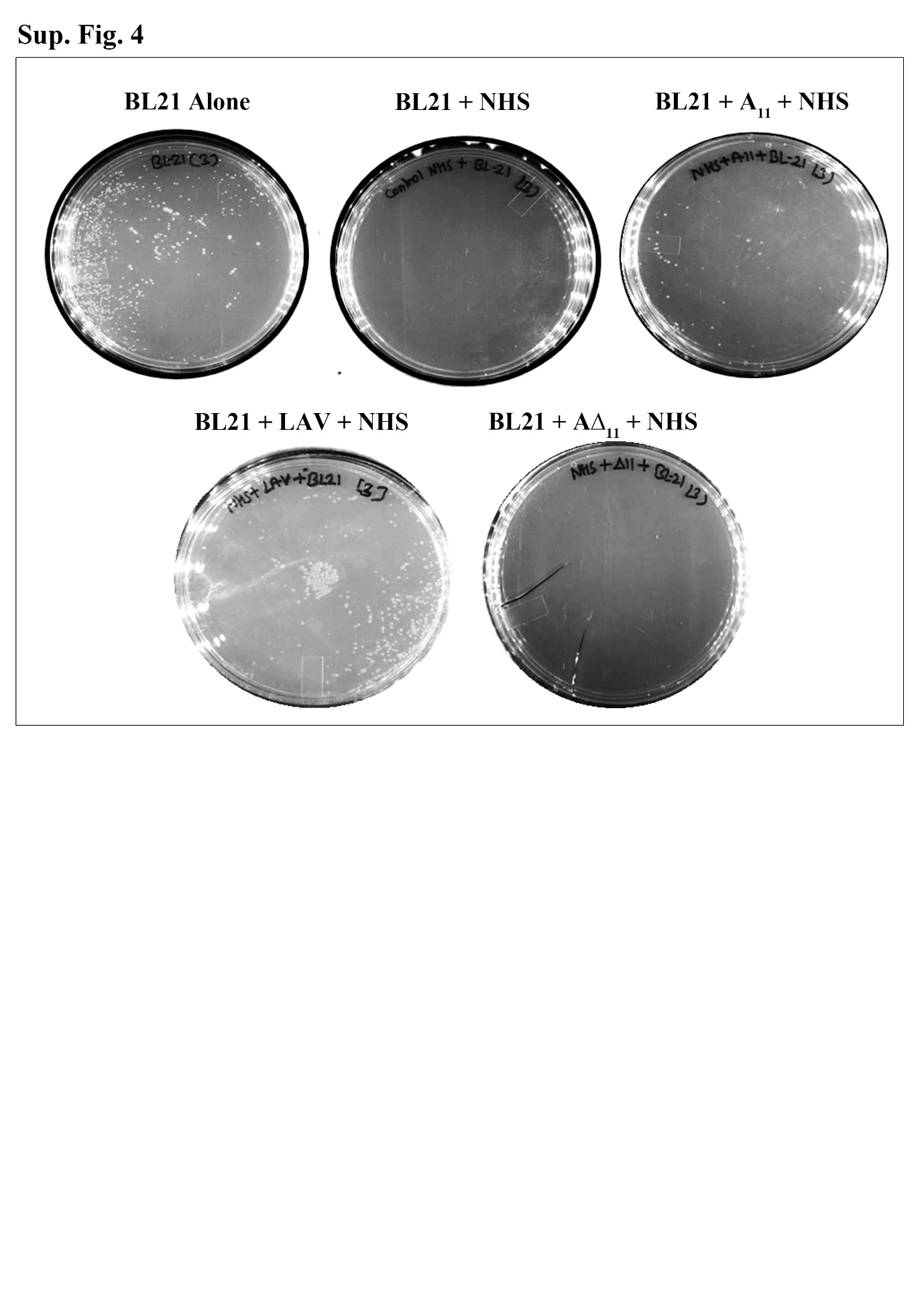
**

**Sup Fig. 4.** **Bacterial survival assay**. Photographic images of survival assay bacterial colonies formed by BL-21 E. coli after the treatment with NHS, which is pre-incubated with LigAvWT, ∆11 and A11.

**Table1. Primers used for RT-PCR**

| S.NO | Gene | Primer Sequence |
| --- | --- | --- |
| 1 | GAPDH | F- GCCTGGAGAAACCTGCC  R- ATACCAGGAAATGAGCTTGACA |
| 2 | IL-4 | F- AGTTGTCATCCTGCTCTTC  R- GTGTTCTTCGTTGCTGTG |
| 3 | CCL2 | F- ACGTGTTGGCTCAGCCAGA  R- ACTACAGCTTCCTTTGGGACACC |
| 4 | COX-2 | F- TCTGGAACATTGTGAACAACATC  R- AAGCTCCTTATTTCCCTTCACAC |
| 5 | iNOS | F- CAGCCCAACAATACAAGATGACCC  R- CAGTTCCGAGCGTCAAAGACCTGC |
| 6 | MCP1 | F- GGAAAAATGGATCCACACCTTGC  R- TCTCTTCCTCCACCACCATGCAG |
| 7 | CXCL10 | F- CATGGTCCTGAGACAAAAGT  R- TGATGACACAAGTTCTTCCA |
| 8 | CXCR4 | F- GAAGTGGGGTCTGGAGACTATG  R- AGGGGAGTGTGATGACAAAGAG |
| 9 | CCR3 | F- CAACTTGGCAATTTCTGACCTG  R- GCAAACACAGCATGGACGATAG |
| 10 | CCR5 | F - ACACTCAGTATCATTTCTGG  R- GGATCAGGCTCAAGATGACC |
| 11 | IFN-γ | F- ACTCAAGTGGCATAGATGTGGAAG  R- GACGCTTATGTTGTTGCTGATGG |
| 12 | IL-1b | F- GCCTTGGGCCTCAAAGGAAAGAATC  R- GGAAGACACAGATTCCATGGTGAAG |
| 13 | IL-10 | F- GCCAGAGCCACATGCTCCTA  R- GATAAGGCTTGGCAACCCAAGTAA |
| 14 | IL-6 | F- TGGAGTCACAGAAGGAGTGGCTAAG  R- TCTGACCACAGTGAGGAATGTCCAC |
| 15 | TNF-α | F- ATAGCTCCCAGAAAAGCAAGC  R- CACCCCGAAGTTCAGTAGACA |

**Table 2. Primers used for domain deletions**

| S.NO | Name | Primer Sequence |
| --- | --- | --- |
| 1 | A∆8 | F- ACCGTCACACAGGCGACTATTGCAGTTGGAAAACAT  R- ATGTTTTCCAACTGCAATAGTCGCCTGTGTGACGGT |
| 2 | A∆9 | F- TCAAATCAGTCCTGTAAAAGATTATCCGTTACCGCA  R- TGCGGTAACGGATAATCTTTTACAGGACTGATTTGA |
| 3 | A∆10 | F- GCAGCGGAACTTATTGAGCAAGTTACTCCGGCTAAA  R- TTTAGCCGGAGTAACTTGCTCAATAAGTTCCGCTGC |
| 4 | A∆11 | F- CAAGTTACTCCGGCTAAATTGAATGTCACTCCAGCG  R- CGCTGGAGTGACATTCAATTTAGCCGGAGTAACTTG |
| 5 | A∆12 | F- ACTCCAGCGCTTCTTCGTCCAGTTACGGTTACGGAA  R- TTCCGTAACCGTAACTGGACGAAGAAGCGCTGGAGT |
| 6 | A∆13 | F- GTTACGGAAAGTGGTATAGTAACTCCAGAAATATTA  R- TAATATTTCTGGAGTTACTATACCACTTTCCGTAAC |
